# Evaluation of 17 microsatellite markers for parentage testing and individual identification of domestic yak (*Bos grunniens*)

**DOI:** 10.7717/peerj.5946

**Published:** 2018-11-12

**Authors:** Jie Pei, Pengjia Bao, Min Chu, Chunnian Liang, Xuezhi Ding, Hongbo Wang, Xiaoyun Wu, Xian Guo, Ping Yan

**Affiliations:** 1 Animal Science Department, Lanzhou Institute of Husbandry and Pharmaceutical Sciences, Chinese Academy of Agricultural Sciences, Lanzhou, Gansu, China; 2 Key Laboratory for Yak Genetics, Breeding, and Reproduction Engineering of Gansu Province, Lanzhou, Gansu, China

**Keywords:** Polled yak, Simple sequence repeats, Forensic genetics, Genetic diversity, Microsatellites, Short tandem repeats, Identification, Parentage testing

## Abstract

**Background:**

Yak (*Bos grunniens*) is the most important domestic animal for people living at high altitudes. Yak ordinarily feed by grazing, and this behavior impacts the accuracy of the pedigree record because it is difficult to control mating in grazing yak. This study aimed to evaluate the pedigree system and individual identification in polled yak.

**Methods:**

A total of 71 microsatellite loci were selected from the literature, mostly from the studies on cattle. A total of 35 microsatellite loci generated excellent PCR results and were evaluated for the parentage testing and individual identification of 236 unrelated polled yaks. A total of 17 of these 35 microsatellite loci had polymorphic information content (PIC) values greater than 0.5, and these loci were in Hardy–Weinberg equilibrium without linkage disequilibrium.

**Results:**

Using multiplex PCR, capillary electrophoresis, and genotyping, very high exclusion probabilities were obtained for the combined core set of 17 loci. The exclusion probability (PE) for one candidate parent when the genotype of the other parent is not known was 0.99718116. PE for one candidate parent when the genotype of the other parent is known was 0.99997381. PE for a known candidate parent pair was 0.99999998. The combined PEI (PE for identity of two unrelated individuals) and PESI (PE for identity of two siblings) were >0.99999999 and 0.99999899, respectively. These findings indicated that the combination of 17 microsatellite markers could be useful for efficient and reliable parentage testing and individual identification in polled yak.

**Discussion:**

Many microsatellite loci have been investigated for cattle paternity testing. Nevertheless, these loci cannot be directly applied to yak identification because the two bovid species have different genomic sequences and organization. A total of 17 loci were selected from 71 microsatellite loci based on efficient amplification, unambiguous genotyping, and high PIC values for polled yaks, and were suitable for parentage analysis in polled yak populations.

## Introduction

Yak (*Bos grunniens*), a member of Bovidae family, has successfully been adapted to the severe cold and low oxygen levels, characteristics of high altitude regions (∼2,500–5,500 m), such as the Himalayas in South-Central Asia, the Qinghai-Tibetan Plateau, Mongolia, and Russia (Wu, 2016). These regions are known for their high elevations, pristine natural environments, and extreme seasonal variations (Mizuno et al., 2015). The ability of yak to survive in such rugged natural environment is due to its varied behavioral, physiological, and genetic adaptations (Barsila et al., 2014; Hu et al., 2012; Qiu et al., 2012). For instance, remarkable reduction of heat production at night when not grazing and increased energy consumption when grazing under free-range conditions enables yaks to save more energy and resist the extremely harsh conditions than other cattle under similar environmental conditions (Ding et al., 2014). Yak can thrive under extreme environmental conditions, such as the Tibetan Plateau where few other animals can survive. In this region, yak have significantly contributed to human life by providing meat, milk, fur, leather, and transportation compared to other animals (Medhammar et al., 2012; Wang et al., 2018).

There are about 13 million domestic yak in China, accounting for approximately 90% of the global yak population. Although there are 18 yak breeds in China, only one breed (Datong) has been generated in a breeding program (Wu, 2016). However, polled yak have been bred for many years at the foot of Ashidan Mountain, as polling reduces the risk of horn-inflicted injury or death among the herdsmen. Accurate genealogical records can help estimate genetic parameters and improve breeding programs, ensuring efficient and effective breeding progression to avoid excessive inbreeding. However, it is difficult to control mating among yaks while grazing. Furthermore, semen samples could be erroneously mislabeled during preparation, and mating records might be misinterpreted because of clerical errors made during artificial insemination. Therefore, accurate yak pedigree records that are compiled by parentage testing and individual identification are essential to the yak breeding process.

Molecular markers can indicate the degree of genetic relatedness between animals, facilitating parentage verification and individual identification (Estoup, Jarne & Cornuet, 2002). Microsatellites refer to short tandem repeats (STRs) or simple sequence repeats (SSRs), and are considered as tracts of DNA motifs ranging from one to 10 nucleotides in length with repeats of 5–50 times (Carneiro Vieira et al., 2016). Microsatellites can be used to develop pedigree animal populations and evaluate animal breeding, supporting genetic improvement by selective breeding (Weising et al., 1997). The application of microsatellites as molecular markers for animal identification and parentage verification produced highly accurate and effective results in both breeding and forensics (Linacre et al., 2011).

Microsatellite marker analysis has been used to verify the parentage in breeding registries and identify individual animals that are linked to a particular database or owner. Microsatellite panels of cattle (Zhao et al., 2017), horse (Kang et al., 2016), sheep (Rosa et al., 2013), dog (Jeong et al., 2015), and parrot (Coetzer et al., 2017) have been well characterized. Parentage control in the beef cattle breeds, Charolais, Limousin, and Preta, in Portugal was assessed using 10 microsatellite markers, and the results revealed a combined exclusion probability (PE) above 0.9995, indicating their ability to exclude a random parent pair (Carolino et al., 2009). The application of 11 microsatellite loci in paternity testing in Yugoslav Pied cattle breed in Serbia revealed a combined PE of 0.999 (Stevanovic et al., 2010). A total of 16 specific microsatellite markers were used to develop a genetic system of meat traceability for several beef cattle breeds, including Japanese Black, Anduo yak, Limousin, Jiaxian Red, Nanyang Yellow, and Luxi Yellow (Zhao et al., 2017). Previous studies reported that microsatellite genotyping was used for population genetics analysis and parentage testing in yak. However, these loci and their primers were originally developed for cattle, and then used directly in yak (Li et al., 2013; Nguyen et al., 2005). Therefore, there is a need for explorative and application-based studies on microsatellite markers or panels that are suitable for accurate individual identification and parentage testing in yak.

Hence, in the present study, we aimed to establish a paternity test and individual identification system for the polled yak. Therefore, the study was expected to (1) calculate the genetic parameters of polled yak microsatellite loci, which have been commonly reported by population genetic studies of cattle and yak; (2) evaluate the application values of the loci with high polymorphic information content (PIC) for parentage testing and individual identification; and (3) explore a multi-loci combination test system for parentage testing and individual identification.

## Materials and Methods

### Marker selection and primer design

The microsatellites used in the present study were selected from the previous reports on cattle breeding based on the following criteria: (a) high PIC; (b) large number of alleles; (c) relatively infrequent null alleles; and (d) homogeneous or approximately homogeneous repeat motifs (Schnabel, Ward & Derr, 2000). Among the 71 bovine microsatellite markers selected, 65 were derived from cattle, and six were exclusive to yak (Table S1). All 71 microsatellites and their flanking sequences were found in the cattle genome, and were searched for in the yak genome. The primers for most loci used in previous studies were not suitable for the yak genome because of low scores. However, the primers used to amplify BM1824, BM2113, BMS2533, ETH121, ETH225, ILSTS008, INRA124, RM099, INRA126, UMN0103, UMN0307, UMN0920, UMN2303, UMN3007, and UMN3008 loci presented relatively high scores, so these loci were not redesigned for yak. The primers used in the present study are listed in Table S1.

### Sample collection

Polled yaks bred via selective breeding were selected from herds in Ashidan Mountain region in Qinghai Province, China. To avoid consanguinity, samples were taken from animals with no genetic relationship. All yaks were handled in strict accordance with good animal practices by following the *Animal Ethics Procedures and Guidelines of the People’s Republic of China*. The present study was approved by the *Animal Administration and Ethics Committee of Lanzhou Institute of Husbandry and Pharmaceutical Sciences of Chinese Academy of Agricultural Sciences* (Permit No. SYXK-2016-0039). Blood was drawn from the jugular veins of 236 unrelated individuals, including 38 sires and 198 dams, and samples were mixed with preservation buffer (containing 1.5 mg mL^−l^ EDTA and 137 mmol L^−l^ NaCl) at a ratio of 5:1. The blood samples were stored at −80 °C in an ultra-cold freezer until DNA extraction.

### DNA extraction and quantification

Genomic DNA was extracted from white blood cells, separated from whole blood, and digested with proteinase K. After digestion, the samples were centrifuged at 5,000×*g* for 2 min, and the resulting supernatant (clear aqueous layer) was transferred to a new test tube. After the addition of 0.5 mL of 10 mg mL^−l^ RNase A, DNA was individually extracted with a phenol:chloroform:isoamyl alcohol mixture (25:24:1) followed by chloroform, precipitated with ethanol, and resuspended in 50 mL TE buffer (10 mM Tris–HCl and 1 mM EDTA, pH 8.0). The extracted DNA was then quantified using a NanoDrop 2000 fluorometer (Thermo Fisher Scientific, Waltham, MA, USA).

### Preliminary primer screening

Unlabeled primer pairs (Table S1) for each microsatellite marker were used for amplification of DNA fragments. PCR was performed with a reaction mixture at a total volume of 20 μL, comprising of 20–50 ng genomic DNA, 10 mM Tris–HCl (pH 9.0), 50 mM KCl, 1.5 mM MgCl_2_, 2.0 mM each dNTP, five ng of bovine serum albumin, and 1.0 U of *Taq*^™^ Hot Start Version polymerase (TaKaRa Bio Inc., Kusatsu, Shiga, Japan). Primer concentrations ranged from 1.0 to 5.5 μM. The thermal cycle parameters were as follows: 5 min at 95 °C; 35 cycles of 30 s at 95 °C, 30 s at 55–58 °C (Table S1), and 20 s at 72 °C; and a final extension step for 5 min at 72 °C. Amplifications were performed on a Veriti 96-Well Thermal Cycler (Applied Biosystems Corp., Foster City, CA, USA). The amplicons were visualized on 2% agarose gels (Gene Tech Co. Ltd, Chai Wan, Hong Kong, People’s Republic of China). Five μL of PCR product were mixed with one μL of loading dye (TaKaRa Bio Inc., Kusatsu, Shiga, Japan) and electrophoresed for 35 min at 85 V. The samples were then visualized using the GelDoc™ XR+ gel imaging system (Bio-Rad Corp., Hercules, CA, USA).

The amplicons with high specificity and high amplification efficiency, whose loci were either non-syntenic or separated by >10 cM (to avoid strong genetic linkage) (Table S2), were manually cut out of agarose gels and forwarded to Invitrogen (Carlsbad, CA, USA) and Thermo Fisher Scientific without prior purification for Sanger sequencing on an ABI3730xl automated sequencer (Applied Biosystems Corp., Foster City, CA, USA) to identify repeat markers. Only the forward primers were used to sequence the target markers.

### Genetic information acquisition

The confirmed primer sequences flanking the microsatellite loci were synthesized with a fluorescent label (FAM™, HEX™, or TAMRA™; Thermo Fisher Scientific, Waltham, MA, USA) attached to the 5′ end of each forward primer. The microsatellites were separately amplified by PCR to identify the highly polymorphic loci. The amplification systems and conditions were similar to those described above for unlabeled primers.

After amplification, one μL of amplified fragment mix was added to 0.5 μL of loading buffer (blue dextran, 50 mg mL^−l^; EDTA, 25 mM) and four μL of deionized formamide, and it was then denatured by incubation for 5 min at 95 °C. An internal size standard (0.5 μL of Thermo ABI 4322682; Thermo Fisher Scientific, Waltham, MA, USA) was added to each sample, and the fluorescently labeled PCR products were then separated by capillary electrophoresis (ABI3730xl Genetic Analyzer; Applied Biosystems Corp., Foster City, CA, USA). The fluorescently labeled DNA fragments were first analyzed with GENESCAN v. 3.7 (Applied Biosystems Corp., Foster City, CA, USA) followed by GENOTYPER v. 3.7 NT (Applied Biosystems Corp., Foster City, CA, USA). The fragments were then automatically sorted according to the internal size standard. The numbers of microsatellite repeat motifs were calculated based on the amplicon lengths (Table S3).

### Genetic information analysis

The genotypic data were initially processed in Microsoft Office Excel 2007, manually checked for errors, and then transformed into input files that were required for subsequent analyses. The performance characteristics (observed heterozygosity (H_O_), expected heterozygosity (H_E_), PIC, estimated null allele frequency (F(null)), Hardy–Weinberg equilibrium (HWE), and linkage disequilibrium) were measured with GENEPOP v. 4.6 (Raymond & Rousset, 1995; Rousset, 2008) and CERVUS v. 3.0.7 (Kalinowski, Taper & Marshall, 2010; Slate, Marshall & Pemberton, 2000).

### Multiplex PCR conditions

Genotyping of 236 yaks for 35 loci (Table 1) produced a core set of 17 loci with high PIC values (Table 2). Four multiplex PCR reactions were assembled, each containing four or five microsatellite markers. The primer sequences and concentrations used in the multiplex PCR reactions are shown in Table 2. The multiplex PCR reactions were performed with reaction mixtures at a total volume of 15 μL, containing 25 ng of genomic DNA, 10 mM Tris–HCl (pH 8.3), 35 mM KCl, 1.8 mM MgCl_2_, 5.0 mM each dNTPs, and 2.5 U of *Taq*^™^ Hot Start Version polymerase (TaKaRa Bio Inc., Kusatsu, Shiga, Japan). Amplifications were performed in a Veriti 96-Well thermal cycler (Applied Biosystems Corp., Foster City, CA, USA) under the following conditions: 95 °C for 5 min; 25 cycles of 95 °C for 30 s, 55–58 °C for 30 s, and 72 °C for 30 s; 10 cycles of 95 °C for 30 s, 53 °C for 30 s, and 72 °C for 30 s; and final extension at 72 °C for 5 min. Genotyping of these microsatellite loci was performed as described above.

**Table 1:** Genetic information for the 35 polymorphic microsatellite loci.

Locus ID	Range (bp)	Repeat motif	N_A_	H_O_	H_E_	PIC	HWE	F(Null)
**BM720**	160–168	AC	5	0.574	0.588	0.541	NS	0.0127
BM1818	239–251	AC	5	0.325	0.329	0.302	NS	0.0031
**BM2113**	115–133	TG	8	0.673	0.662	0.623	NS	0.0067
BM2943	253	AC	1	0	0	0	ND	ND
CSSM013	156–160	TG	3	0.294	0.309	0.272	NS	0.0180
CSSM029	182–194	AC	6	0.517	0.466	0.417	NS	0.0623
CSSM033	309–317	TG	3	0.137	0.130	0.126	ND	0.0261
**CSSM036**	157–179	TG	10	0.677	0.693	0.646	NS	0.0116
CSSM041	128–134	TG	4	0.457	0.428	0.402	NS	0.0427
CSSME070	249–259	AC	5	0.513	0.526	0.412	NS	0.0093
**HAUT24**	217–231	AC	8	0.628	0.633	0.572	NS	0.0059
HEL5	214–228	TG	6	0.182	0.181	0.174	ND	0.0055
**HEL6**	247–265	GT	8	0.686	0.754	0.721	NS	0.0427
HEL10	171–177	TG	4	0.091	0.502	0.417	***	0.7025
ILSTS006	162–170	GT	4	0.453	0.448	0.374	NS	0.0095
ILSTS008	172–184	AC	6	0.577	0.550	0.446	NS	0.0257
**ILSTS028**	261–293	GT	11	0.806	0.838	0.815	NS	0.0194
ILSTS030	150–156	GT	4	0.106	0.129	0.123	ND	0.1033
**INRA005**	186–202	CA	5	0.607	0.604	0.529	NS	0.0058
**INRA023**	184–204	TG	10	0.753	0.780	0.752	NS	0.0162
INRA035	104	TG	1	0	0	0	ND	ND
**INRA037**	296–312	CA	8	0.688	0.770	0.730	NS	0.0576
**INRA063**	175–187	TG	7	0.604	0.642	0.585	NS	0.0317
MGTG4B	245–255	AC	5	0.196	0.273	0.254	**	0.1631
MM12	109–121	GT	4	0.085	0.087	0.084	ND	0.0242
**POTCHA**	128–148	CA	8	0.573	0.585	0.540	NS	0.0227
RM099	233	CA	1	0	0	0	ND	ND
**SPS115**	231–261	CA	12	0.782	0.806	0.780	NS	0.0146
TGLA57	253–263	GT	6	0.348	0.340	0.322	NS	0.0120
**TGLA126**	209–223	TG	8	0.587	0.690	0.639	NS	0.0819
**TGLA227**	210–222	AC	5	0.611	0.617	0.538	NS	0.0015
**YAK07**	323–339	TG	9	0.63	0.621	0.589	NS	0.0104
**YAK08**	321–343	CA	8	0.698	0.676	0.612	NS	0.0218
**YAK11**	306–314	GT	7	0.679	0.666	0.597	NS	0.0096
YAK12	259–279	GT	8	0.549	0.522	0.406	NS	0.0269

**Notes:**

Font bold, core microsatellite loci are indicated in bold.

Range, range of allele sizes; Repeat motif, repeat motif of microsatellites; N_A_, number of alleles found; H_O_, observed heterozygosity; H_E_, expected heterozygosity; PIC, polymorphism information content; HWE, departure from Hardy–Weinberg equilibrium; NS, not significant; ND, not done; F(Null), estimated null allele frequency.

** Significant at the 1% level;

*** Significant at the 0.1% level.

**Table 2:** Detailed primer information for the core microsatellite loci identified in the present study.

Locus name	Forward primer (5′→3′)	Reverse primer (5′→3′)	C [mM]	Ta (°C)	PMP	PML	Fluoro	RM	EP1	EP2	EPP	EI	ESI	CL
BM720	GAAATCAACAAGTTCCAATCCTG	ATCTCATTCTTGTGTCATGGATGA	3.5	56	3	2	6-FAM	(AC)	0.189	0.353	0.532	0.783	0.489	13
BM2113	GCTGCCTTCTACCAAATACCC	CTTCCTGAGAGAAGCAACACC	1.9	55	1	1	6-FAM	(TG)	0.261	0.439	0.636	0.847	0.542	2
CSSM036	GATAACTCAACCACACGTCT	AAGAAGTACTGGTTGCCAATCG	2.8	56	2	1	6-FAM	(TG)	0.283	0.456	0.644	0.859	0.561	27
HAUT24	CTCTGCCTTTGTCCCTGTCT	CCAAACCCCCTACCCACA	5.7	56	2	1	TAMARA	(AC)	0.221	0.377	0.552	0.805	0.517	22
HEL6	GGACACGACTGAGCAAGTAACA	GCTTTGGCAGGCAGATACAT	4.0	56	1	1	HEX	(GT)	0.367	0.549	0.743	0.907	0.603	1
ILSTS028	AGAAGAGTGTACCTCCTCCCAC	TCCAGATTTTGTACCAGACCAT	4.6	56	1	1	TAMARA	(GT)	0.503	0.673	0.847	0.953	0.656	11
INRA005	CTTCAGGCATACCCTACACCA	GGGGAATCTGTGGAGGAGTT	8.3	56	4	2	6-FAM	(CA)	0.190	0.328	0.483	0.768	0.494	12
INRA023	ATTTCCCTTCTGACTGGTACTTC	GTGTCCCTCCTCTAATCCCTAA	3.0	55	3	2	HEX	(TG)	0.408	0.589	0.782	0.924	0.620	3
INRA037	GCTACAATCCAGACTGAGCACG	GACACGGCTTAGCGACTGAA	3.1	57	3	2	HEX	(CA)	0.370	0.549	0.731	0.909	0.611	10
INRA063	AAACCACAGAAATGCTTGGAAG	ATTTGCACAAGCTAAATCTAACAA	3.6	56	1	1	TAMARA	(TG)	0.228	0.390	0.566	0.816	0.524	18
POTCHA	ATGCCAACTTTTCCCATCACT	GTAAACACAGTTCCCTGGAGAGA	3.5	56	2	1	HEX	(CA)	0.192	0.357	0.540	0.783	0.488	15
SPS115	AAAGTGACACAACAGCTTCACC	ACCGAGTGTCCTAGTTTGGC	4.6	55	4	2	TAMARA	(CA)	0.452	0.628	0.814	0.938	0.636	15
TGLA126	ATGAGAGAGGCTTCTGGGATG	CTTCACCATTGGACCACGAG	3.7	56	4	2	HEX	(TG)	0.272	0.444	0.625	0.854	0.558	20
TGLA227	CAAAGGAGCATAACTTTACAGCA	AGCCCTAACCATTGGACAGC	4.9	57	3	2	TAMARA	(AC)	0.200	0.337	0.494	0.775	0.501	18
YAK07	TAACAAAGCTGCTGGGAACAT	CGGAGTCACTTTCCTCACCTAT	2.4	56	4	2	HEX	(TG)	0.230	0.412	0.615	0.825	0.516	1
YAK08	ACTGGAGTAGGTTGCCCTGC	CCTGGCTTGGTCCTGTCTCT	3.8	56	2	1	HEX	(CA)	0.247	0.405	0.572	0.831	0.545	6
YAK11	TCCCCTCACTCCTCATTGGT	TGCAGGCAGTTTCTTACCAGT	4.4	56	3	2	TAMARA	(GT)	0.233	0.387	0.548	0.820	0.537	1
Combined									0.99718116	0.99997381	0.99999998	>0.99999999	0.99999899	

**Note:**

C, concentration of primers; Ta, annealing temperature; PMP, panel numbers for multiplex PCR; PML, panel numbers for multiplex loading; Fluoro, fluorescent dye; RM, repeat motif of microsatellites; PE1, the average probability that the set of loci will exclude an unrelated candidate parent from parentage of an arbitrary offspring when the genotype of the other parent is unknown; PE2, the average probability that the set of loci will exclude an unrelated candidate parent from parentage of an arbitrary offspring when the genotype of the other parent is known; PEP, the average probability that the set of loci will exclude a pair of unrelated candidate parents from parentage of an arbitrary offspring; PEI, the average probability that the set of loci will differentiate between two randomly selected individuals; PESI, the average probability that the set of loci will differentiate between two randomly selected full siblings; CL, locations of microsatellite loci on cattle chromosomes.

### Parentage testing and individual identification

PE values for parentage testing were obtained using a likelihood-based method based on genotypic information. Five types of PEs were calculated for the set of loci in CERVUS v. 3.0.7. PE1 was defined as the average probability of excluding an unrelated candidate parent of an arbitrary offspring when the genotype of the other parent is unknown. PE2 was defined as the average probability of excluding an unrelated candidate parent of an arbitrary offspring when the genotype of the other parent is known. PEP was defined as the average probability of excluding a pair of unrelated candidate parents of an arbitrary offspring. PEI was defined as the average probability of differentiating two randomly selected individuals. PESI was defined as the average probability of differentiating two randomly selected full siblings (Kalinowski, Taper & Marshall, 2010; Slate, Marshall & Pemberton, 2000).

## Results

### Microsatellite loci characteristics

After preliminary screening, a total of 35 microsatellite loci with the highest primer specificity among the 71 microsatellite loci were selected for further analysis. The number of alleles, allele size range, H_O_, H_E_, PIC, F(null), and HWE of the 35 microsatellite loci of polled yaks are presented in Table 1. Three monomorphic loci, namely BM2943, INRA035, and RM099, were identified. The remaining 32 loci were polymorphic. The number of alleles per locus ranged from 3 (CSSM013 and CSSM033) to 12 (SPS115), and PIC values ranged from 0.084 (MM12) to 0.815 (ILSTS028). Four of these 32 polymorphic loci had low PIC values (i.e., <0.25), 11 had moderate PIC values (0.25–0.50), and 17 loci had high PIC values (>0.50) (Table 1).

The 17 genetic markers with high PIC values were reproducible, informative, and locus-specific for parentage testing and individual identification. The number of alleles ranged from five (BM720, INRA005, TGLA227) to 12 (SPS115). Heterozygosity values ranged from 0.573 (POTCHA) to 0.806 (ILSTS028), with an average of 0.662. Deviations between H_O_ and H_E_ ranged from 0.003 (INRA005) to 0.103 (TGLA126). The 17 loci had an average PIC values of 0.636, ranging from 0.529 (INRA005) to 0.815 (ILSTS028). The null allele frequency ranged from 0.0015 (TGLA227) to 0.0819 (TGLA126) (Table 1).

### Hardy–Weinberg and linkage disequilibrium tests

The results of HWE tests of the 17 microsatellite loci indicated no significant differences (*P* > 0.05) (Table 1). Therefore, the 17 loci with high PIC values (>0.5) were selected for yak paternity testing. Linkage disequilibrium within polled yaks resulted in 136 comparisons, and no interlocus disequilibrium was detected between loci on the same chromosome (Table S6).

### Multiplex amplification and loading

**Figure 1:**
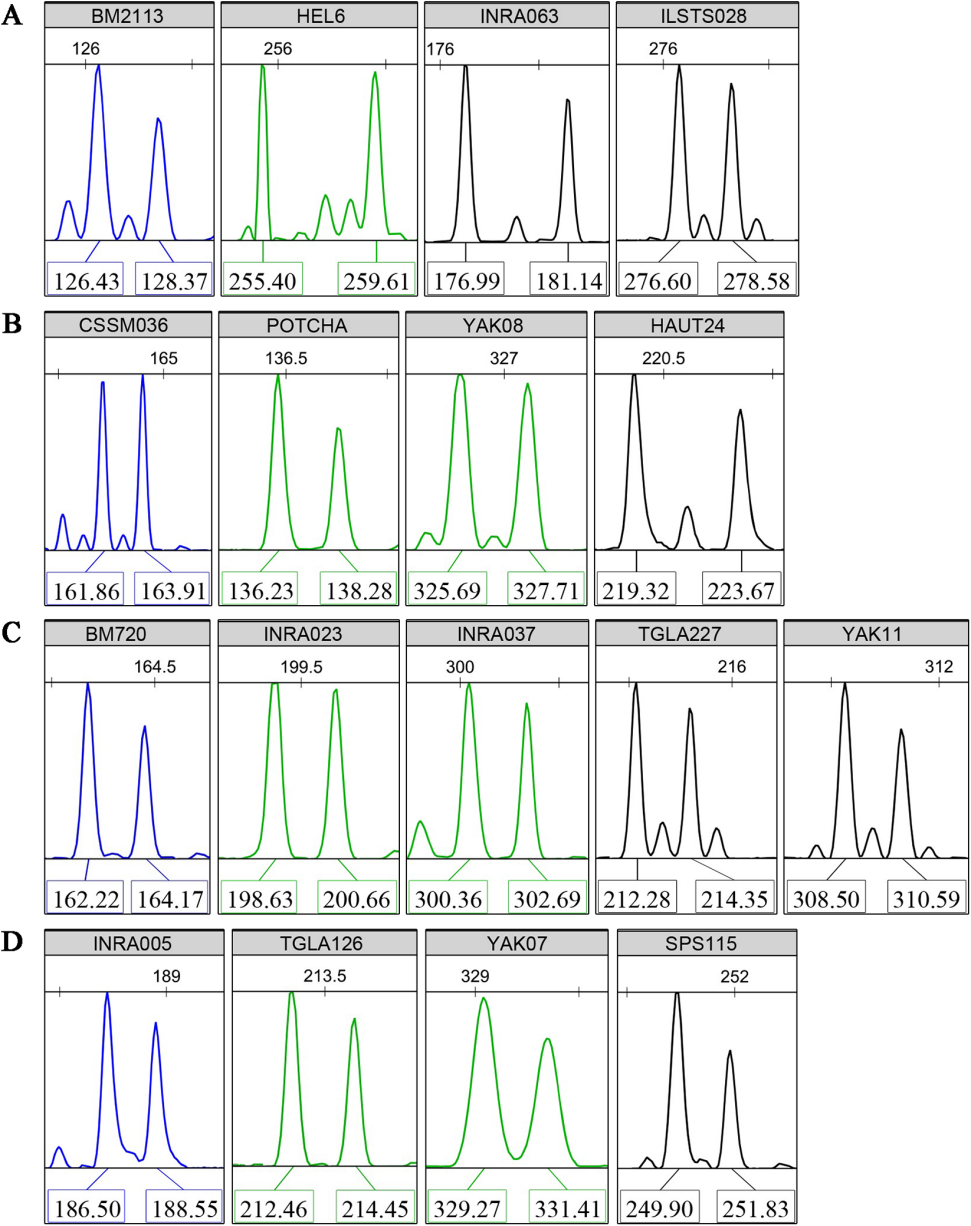
Typical fluorescence signal s of detections for the core 17 microsatellite loci. (A) Panel 1, (B) Panel 2, (C) Panel 3, (D) Panel 4. The “Panels” stand for the multiplex PCR groups. The colors of fluorochrome 6-FAM, HEX, and TAMARA are showed by blue, green, and black, respectively. The numbers above the sharp peaks represent length scales based on the internal size standard (bp). The numbers below the sharp peaks represent fragment lengths of PCR amplifications (bp).

Multiplex PCR reactions were organized to co-amplify four groups of four to five loci with high PIC values. Non-overlapping allele lengths that presented the same fluorescent color label were selected. Typical fluorescence signals of the core 17 microsatellite loci are shown in Fig. 1. Using the three available fluorescent colors, we multiplex-loaded and scored two groups of eight or nine loci in each run.

### Parentage inference

The exclusion probabilities were calculated from the allele frequencies based on the underlying assumptions of HWE. The PE1 values of the 17 core loci ranged from 0.189 (BM720) to 0.503 (ILSTS028), with an average of 0.285. The average PE2 values of the 17 markers was 0.451, and values ranged from 0.328 (INRA005) to 0.673 (ILSTS028). Regarding the 17 core loci, the combined PE1 and PE2 values were 0.99718116 and 0.99997381, respectively. For the putative parents, the combined PEP value was 0.99999998. Combined PEI and PESI values were >0.99999999 and 0.99999899, respectively (Table 2). Therefore, the PE values indicated that the discriminatory power of the 17 loci was high.

## Discussion

Previous studies indicated that 4.3% of annual losses with regard to genetic gain during dairy breeding were caused by pedigree errors (10%), compared to simulation analysis of accurate paternity determination data (Israel & Weller, 2000). In fact, the pedigree error rate in yaks was high due to incorrect paternity as yaks feed primarily by grazing, thwarting parentage attribution. Additionally, clerical and insemination errors and fading ink on artificial insemination records and labels might contribute to sample mixing, thereby, leading to pedigree errors. Therefore, it is necessary to identify and correct the pedigree through parentage testing and individual identification. These practices that aim at genetic improvement of yak are essential for generating reliable breeding programs. Several reports have been published on the use of microsatellite markers for cattle identification (Sharma et al., 2015; Zhao et al., 2017), but the performance characteristics of yak identification panels have not yet been established. Parentage testing for yak breeding increases the profitability by improving the efficiency of selective breeding programs.

The most commonly used methods of livestock identification and parentage verification rely on microsatellites (Jan & Fumagalli, 2016; Jeong et al., 2015; Wang et al., 2017). However, single nucleotide polymorphisms (SNPs) have been applied in the identification and parentage verification of swine (*Sus scrofa*) and cattle (*B. taurus*) (Eggen, 2012; Rohrer, Freking & Nonneman, 2007). A recent study debated on the use of SNPs instead of microsatellites for parentage verification (Kaiser et al., 2017). However, at least 200 SNPs should be used for parentage testing to reduce false-negative results, and at least 700 SNPs are required to completely eliminate false positives (Strucken et al., 2016). In addition, parentage analysis based on SNPs has few predictable statistical problems that must be considered carefully and evaluated appropriately before substituting the classical STRs approach (Amorim & Pereira, 2005). For these reasons, microsatellites are preferred over SNPs for parentage testing.

Most of the microsatellite markers used for cattle identification and parentage verification are dimeric repeat motifs (Carolino et al., 2009; Stevanovic et al., 2010; Van De Goor, Panneman & Van Haeringen, 2009; Zhao et al., 2017). Similarly, all microsatellite markers used in the present study were dimeric. Repeat patterns have advantages as well as disadvantages. Dimeric microsatellites might have mutations or stutter bands that present allele interpretation errors (Walsh, Fildes & Reynolds, 1996). On the contrary, during PCR conduction, trimeric, tetrameric, and pentameric repeat motifs demonstrated lower stutter slippage efficiency than dimeric microsatellites (Gill, Curran & Elliot, 2005), and they ensured clear peak discrimination. On the other hand, an appropriate mutation rate might be beneficial for the verification of multigenerational parentage. This is because mutations might occur between an ancestor and a descendant’s assumed father/mother, but not between an assumed father/mother and offspring, thus allowing the identification of true father/mother. For trimeric, tetrameric, pentameric, and hexameric microsatellites, gaps occurring during the sequence variant visualization within the repeat units can result in larger bin sizes than those of dimeric microsatellites (Gill et al., 2000). However, if the span of the sequence variants is too wide, it becomes increasingly difficult to confine the microsatellite markers within a fluorochrome to a single multiplex PCR system. Furthermore, multimeric repeats can also be compound. All of the microsatellite markers used in the present study were dimeric containing homogeneous repeat motifs, therefore, some markers can be labeled as one fluorochrome in a single multiplex PCR with unambiguous genotyping.

In order to use them in paternity testing and individual identification, microsatellite loci must have rare null alleles, and be at HWE, and the gametic association (linkage disequilibrium) should be absent. Null alleles are not amplified to detectable levels via PCR because of mutations at primer binding sites (Kline et al., 2011). The frequency of null alleles is mainly estimated by Mendelian incompatibilities (Strucken et al., 2016) and by comparing the observed and expected number of homozygotes at a locus (Dąbrowski et al., 2015). This fact must be considered when performing genotyping for parentage testing and when there is an apparent opposite homozygosity between parent and offspring. In the present study, the estimated null allele frequencies remained the highest for HEL10 (F(Null) = 0.703) and MGTG4B (F(Null) = 0.163). Therefore, these loci were excluded from the core set used for parental identification.

The formulae used to estimate the exclusion probabilities assume random mating, random association between alleles of different loci, and allele frequencies consistent with HWE. The 17 loci that were selected as core microsatellite markers were at HWE (Table 1), indicating that they can be used to calculate PE values.

The microsatellites used in cattle parentage testing could not be directly applied to yak identification due to uncertainty of whether the primers used for cattle would produce the desired results in yak. In the present study, the microsatellite primers were designed based on the yak gene sequences, and were then tested by PCR and electrophoresis. In addition, the allelic frequencies differed between the cattle and yak. Even among cattle breeds, the microsatellite PIC values differed (Mao et al., 2008), and the same might be applied to yak breeds (Zhang et al., 2008). Thus, while determining the PE values of parentage testing and individual identification for a new breed, allele frequencies and PIC values should be calculated de novo.

The microsatellite loci with PIC value > 0.5 were selected for the identification panel. Nevertheless, the PIC values of these loci were still moderately lower than those used for cattle testing (Stevanovic et al., 2010; Vohra et al., 2017). Therefore, a high number of microsatellite markers were screened in the present study, and a set of 17 microsatellite markers proved to be sufficient for determining PE values. The combined exclusion probabilities of wrongly assigned sires were 99.718116%, 99.997381%, and 99.999998% for PE1, PE2, and PEP, respectively. Similar results were reported in Angeln dairy cattle, with 16 microsatellites and approximately 99.9% PE1 (Sanders, Bennewitz & Kalm, 2006). The PEP value calculated for the set of 17 microsatellites for parentage testing in the present study was 99.997381%, which was marginally higher than that obtained for Swiss yaks (99.5%) using 13 STR markers (Nguyen et al., 2005). Therefore, the set of microsatellite loci used in the present study significantly contributed to parental identification in the polled yak population.

We used several mapped cattle microsatellites to develop the sets of yak loci that were suitable for multiplex PCR amplification, and multiplex loading was conducted in a single run to reduce human errors, typing cost, and time. Nevertheless, selecting markers for a universal panel depends on the balance among the required panel accuracy, amplicon length, and ability to undergo a successful multiplex reaction. Multiplex PCR amplifications are technically more difficult than their single-locus counterparts, but they are less likely to transfer across species than single-locus amplifications. Several multiplex PCR and loading optimization methods have been investigated for parentage testing of cattle. Nevertheless, these methods cannot be directly applied to yak identification. Therefore, we developed four multiplex amplifications (each containing four or five loci) and two multiplex loads (each containing eight or nine loci) running in two gel lanes. The main advantage of this system is that the allele length ranges do not overlap within the same fluorochrome.

Hence, we proposed that a combination of 17 microsatellites can yield a polled yak panel with enhanced processing efficiency, reliability, and utility. Moreover, this system uses the standard genotyping methods of DNA fragment analysis technology. Combined with likelihood-based parentage testing, these 17 markers will help improve breeding programs and accurately determine polled yak pedigrees. If this system is used to identify polled yaks that are not the descendants of the expected breeding male yak, breeders can eliminate them from the breeding group to ensure genetic purity and breed improvement. On the other hand, if the semen samples of high-grade male yaks are mixed with those of other males, the detection system can use DNA from semen and blood of the high-grade male yak for identification. Multiplex systems can also be used to rapidly assess the history, structure, and diversity of the breeding population, and these systems can reconstruct relationships among breeds. Furthermore, these multiplex systems might also be applied to other yak breeds with gene frequencies that are similar to that of the population tested in the present study.

Although the core set of microsatellite loci presented here was meaningful for yak parentage testing, this methodology still has the following limitations: (a) Since different yak breeds have different microsatellite genotypes, this core set was only suitable for parentage testing of polled yak; (b) The PIC values of the microsatellite loci are not sufficiently high to reduce the number of loci, thus avoiding low PE values; and (c) The number of panels for multiplex PCR reactions and multiplex loading still remained too high, leading to time-consuming and high cost. Therefore, a large number of microsatellites with high PIC values should be obtained for different yak breeds to develop efficient parentage test systems, with higher PE values and fewer markers. Furthermore, microsatellites should be suitable to each specific yak breed, and markers should be screened using single multiplex PCR reactions or multiplex loading.

## Conclusions

A set of 17 microsatellite markers, which were assembled into four multiplex PCR reaction systems and genotyped in two multiplex loading systems, were identified and evaluated. The high variability displayed by these microsatellite loci demonstrated that highly precise genotyping panels might be used for individual genotyping, parentage verification, and individual identification. The microsatellites reported in this study could also be used to evaluate yak population structure, history, and diversity, which subsequently aids the genetic improvement of domestic yak.

## Supplemental Information

10.7717/peerj.5946/supp-1 Supplemental Information 1 Table S1. Primer information for the 71 polymorphic microsatellite loci designed according to the yak genome sequences.

10.7717/peerj.5946/supp-2 Supplemental Information 2 Table S2. Locations of the 35 polymorphic microsatellite loci used for allele frequency analysis in cattle chromosome.

10.7717/peerj.5946/supp-3 Supplemental Information 3 Table S3. Repeat number assignment for the 35 loci based on amplicon length.

10.7717/peerj.5946/supp-4 Supplemental Information 4 Table S4. Genotypes of the 35 loci in the 236 polled yaks.

10.7717/peerj.5946/supp-5 Supplemental Information 5 Table S5. Genotype distributions of the 35 loci.

10.7717/peerj.5946/supp-6 Supplemental Information 6 Table S6. Linkage disequilibrium test for the 17 loci in the core set.

## Additional Information and Declarations

### Data Availability

The following information was supplied regarding data availability: The raw data are provided in the Supplemental Files.

## References

[ref-1] Amorim A, Pereira L (2005). Pros and cons in the use of SNPs in forensic kinship investigation: a comparative analysis with STRs. Forensic Science International.

[ref-2] Barsila SR, Kreuzer M, Devkota NR, Ding L, Marquardt S (2014). Adaptation to Himalayan high altitude pasture sites by yaks and different types of hybrids of yaks with cattle. Livestock Science.

[ref-3] Carneiro Vieira ML, Santini L, Diniz AL, Munhoz CdF (2016). Microsatellite markers: what they mean and why they are so useful. Genetics and Molecular Biology.

[ref-4] Carolino I, Sousa CO, Ferreira S, Carolino N, Silva FS, Gama LT (2009). Implementation of a parentage control system in Portuguese beef-cattle with a panel of microsatellite markers. Genetics and Molecular Biology.

[ref-5] Coetzer WG, Downs CT, Perrin MR, Willows-Munro S (2017). Testing of microsatellite multiplexes for individual identification of Cape Parrots (*Poicephalus robustus*): paternity testing and monitoring trade. PeerJ.

[ref-6] Dąbrowski M, Bornelöv S, Kruczyk M, Baltzer N, Komorowski J (2015). “True” null allele detection in microsatellite loci: a comparison of methods, assessment of difficulties and survey of possible improvements. Molecular Ecology Resources.

[ref-7] Ding LM, Wang YP, Brosh A, Chen JQ, Gibb MJ, Shang ZH, Guo XS, Mi JD, Zhou JW, Wang HC, Qiu Q, Long RJ (2014). Seasonal heat production and energy balance of grazing yaks on the Qinghai-Tibetan plateau. Animal Feed Science and Technology.

[ref-8] Eggen A (2012). The development and application of genomic selection as a new breeding paradigm. Animal Frontiers.

[ref-9] Estoup A, Jarne P, Cornuet JM (2002). Homoplasy and mutation model at microsatellite loci and their consequences for population genetics analysis. Molecular Ecology.

[ref-10] Gill P, Curran J, Elliot K (2005). A graphical simulation model of the entire DNA process associated with the analysis of short tandem repeat loci. Nucleic Acids Research.

[ref-11] Gill P, Whitaker J, Flaxman C, Brown N, Buckleton J (2000). An investigation of the rigor of interpretation rules for STRs derived from less than 100 pg of DNA. Forensic Science International.

[ref-12] Hu Q, Ma T, Wang K, Xu T, Liu J, Qiu Q (2012). The Yak genome database: an integrative database for studying yak biology and high-altitude adaption. BMC Genomics.

[ref-13] Israel C, Weller J (2000). Effect of misidentification on genetic gain and estimation of breeding value in dairy cattle populations. Journal of Dairy Science.

[ref-14] Jan C, Fumagalli L (2016). Polymorphic DNA microsatellite markers for forensic individual identification and parentage analyses of seven threatened species of parrots (family Psittacidae). PeerJ.

[ref-15] Jeong H, Choi B-H, Lee H-E, Gim J-A, Kim H-S (2015). Microsatellite analysis of genetic variation and structure in Korean and exotic dog breeds. Genes & Genomics.

[ref-16] Kaiser S, Taylor S, Chen N, Sillett T, Bondra E, Webster M (2017). A comparative assessment of SNP and microsatellite markers for assigning parentage in a socially monogamous bird. Molecular Ecology Resources.

[ref-17] Kalinowski ST, Taper ML, Marshall TC (2010). Revising how the computer program CERVUS accommodates genotyping error increases success in paternity assignment. Molecular Ecology.

[ref-18] Kang SW, Lee SY, Chio DH, Kang HJ, Hu MB, Yang YJ (2016). Statistical analysis of alleles in 4703 thoroughbred racing horses using fifteen microsatellite DNA markers. Journal of Animal Science.

[ref-19] Kline MC, Hill CR, Decker AE, Butler JM (2011). STR sequence analysis for characterizing normal, variant, and null alleles. Forensic Science International: Genetics.

[ref-20] Li D, Chai Z, Ji Q, Zhang C, Xin J, Zhong J (2013). Genetic diversity of DNA microsatellite for Tibetan yak. Hereditas.

[ref-21] Linacre A, Gusmao L, Hecht W, Hellmann AP, Mayr WR, Parson W, Prinz M, Schneider PM, Morling N (2011). ISFG: recommendations regarding the use of non-human (animal) DNA in forensic genetic investigations. Forensic Science International: Genetics.

[ref-22] Mao Y, Chang H, Yang Z, Zhang L, Xu M, Chang G, Sun W, Song G, Ji D (2008). The analysis of genetic diversity and differentiation of six Chinese cattle populations using microsatellite markers. Journal of Genetics and Genomics.

[ref-23] Medhammar E, Wijesinha-Bettoni R, Stadlmayr B, Nilsson E, Charrondiere UR, Burlingame B (2012). Composition of milk from minor dairy animals and buffalo breeds: a biodiversity perspective. Journal of the Science of Food and Agriculture.

[ref-24] Mizuno S, Ishizaki T, Toga H, Sakai A, Isakova J, Taalaibekova E, Baiserkeev Z, Kojonazarov B, Aldashev A (2015). Endogenous asymmetric dimethylarginine pathway in high altitude adapted yaks. BioMed Research International.

[ref-25] Nguyen T, Genini S, Ménétrey F, Malek M, Vögeli P, Goe M, Stranzinger G (2005). Application of bovine microsatellite markers for genetic diversity analysis of Swiss yak (*Poephagus grunniens*). Animal Genetics.

[ref-26] Qiu Q, Zhang G, Ma T, Qian W, Wang J, Ye Z, Cao C, Hu Q, Kim J, Larkin DM, Auvil L, Capitanu B, Ma J, Lewin HA, Qian X, Lang Y, Zhou R, Wang L, Wang K, Xia J, Liao S, Pan S, Lu X, Hou H, Wang Y, Zang X, Yin Y, Ma H, Zhang J, Wang Z, Zhang Y, Zhang D, Yonezawa T, Hasegawa M, Zhong Y, Liu W, Zhang Y, Huang Z, Zhang S, Long R, Yang H, Wang J, Lenstra JA, Cooper DN, Wu Y, Wang J, Shi P, Liu J (2012). The yak genome and adaptation to life at high altitude. Nature Genetics.

[ref-27] Raymond M, Rousset F (1995). GENEPOP (version 1.2): population genetics software for exact tests and ecumenicism. Journal of Heredity.

[ref-28] Rohrer GA, Freking BA, Nonneman D (2007). Single nucleotide polymorphisms for pig identification and parentage exclusion. Animal Genetics.

[ref-29] Rosa AJM, Sardina MT, Mastrangelo S, Tolone M, Portolano B (2013). Parentage verification of Valle del Belice dairy sheep using multiplex microsatellite panel. Small Ruminant Research.

[ref-30] Rousset F (2008). GENEPOP’007: a complete re-implementation of the GENEPOP software for Windows and Linux. Molecular Ecology Resources.

[ref-31] Sanders K, Bennewitz J, Kalm E (2006). Wrong and missing sire information affects genetic gain in the Angeln dairy cattle population. Journal of Dairy Science.

[ref-32] Schnabel RD, Ward TJ, Derr JN (2000). Validation of 15 microsatellites for parentage testing in North American bison, Bison bison and domestic cattle. Animal Genetics.

[ref-33] Sharma R, Kishore A, Mukesh M, Ahlawat S, Maitra A, Pandey A, Tantia M (2015). Genetic diversity and relationship of Indian cattle inferred from microsatellite and mitochondrial DNA markers. BMC Genetics.

[ref-34] Slate J, Marshall T, Pemberton J (2000). A retrospective assessment of the accuracy of the paternity inference program CERVUS. Molecular Ecology.

[ref-35] Stevanovic J, Stanimirovic Z, Dimitrijevic V, Maletic M (2010). Evaluation of 11 microsatellite loci for their use in paternity testing in Yugoslav Pied cattle (YU Simmental cattle). Czech Journal of Animal Science.

[ref-36] Strucken EM, Lee SH, Lee HK, Song KD, Gibson JP, Gondro C (2016). How many markers are enough? Factors influencing parentage testing in different livestock populations. Journal of Animal Breeding and Genetics.

[ref-37] Van De Goor LHP, Panneman H, Van Haeringen WA (2009). A proposal for standardization in forensic bovine DNA typing: allele nomenclature of 16 cattle-specific short tandem repeat loci. Animal Genetics.

[ref-38] Vohra V, Sodhi M, Niranjan SK, Mishra AK, Chopra A, Kumar M, Joshi BK (2017). Characterization of rare migratory cattle and evaluation of its phylogeny using short-tandem-repeat-based markers. Journal of Applied Animal Research.

[ref-39] Walsh PS, Fildes NJ, Reynolds R (1996). Sequence analysis and characterization of stutter products at the tetranucleotide repeat locus vWA. Nucleic Acids Research.

[ref-40] Wang G, Chen S, Chao T, Ji Z, Hou L, Qin Z, Wang J (2017). Analysis of genetic diversity of Chinese dairy goats via microsatellite markers. Journal of Animal Science.

[ref-41] Wang L-L, Yu Q-L, Han L, Ma X-L, Song R-D, Zhao S-N, Zhang W-H (2018). Study on the effect of reactive oxygen species-mediated oxidative stress on the activation of mitochondrial apoptosis and the tenderness of yak meat. Food Chemistry.

[ref-42] Weising K, Winter P, Huttel B, Kahl G (1997). Microsatellite markers for molecular breeding. Journal of Crop Production.

[ref-43] Wu J (2016). The distributions of Chinese yak breeds in response to climate change over the past 50 years. Animal Science Journal.

[ref-44] Zhang G, Chen W, Xue M, Wang Z, Chang H, Han X, Liao X, Wang D (2008). Analysis of genetic diversity and population structure of Chinese yak breeds (*Bos grunniens*) using microsatellite markers. Journal of Genetics and Genomics.

[ref-45] Zhao J, Zhu C, Xu Z, Jiang X, Yang S, Chen A (2017). Microsatellite markers for animal identification and meat traceability of six beef cattle breeds in the Chinese market. Food Control.

